# Synthesis of carbon nanowalls from a single-source metal-organic precursor

**DOI:** 10.3762/bjnano.9.181

**Published:** 2018-06-29

**Authors:** André Giese, Sebastian Schipporeit, Volker Buck, Nicolas Wöhrl

**Affiliations:** 1 Faculty of Physics and CENIDE, University Duisburg Essen, Carl-Benz-Straße 199, 47057 Duisburg, Germany

**Keywords:** aluminium acetylacetonate, carbon nanowalls, growth zones, ICP PECVD, metal-organic precursor

## Abstract

In this work, the deposition of carbon nanowalls (CNWs) by inductively coupled plasma enhanced chemical vapor deposition (ICP-PECVD) is investigated. The CNWs are electrically conducting and show a large specific surface area, which is a key characteristic to make them interesting for sensors, catalytic applications or energy-storage systems. It was recently discovered that CNW films can be deposited by the use of the single-source metal-organic precursor aluminium acetylacetonate. This precursor is relatively unknown in combination with the ICP-PECVD deposition method in literature and, thus, based on our previous publication is further investigated in this work to better understand the influence of the various deposition parameters on the growth. Silicon, stainless steel, nickel and copper are used as substrate materials. The CNWs deposited are characterized by scanning electron microscopy (SEM), Raman spectroscopy and Auger electron spectroscopy (AES). The combination of bias voltage, the temperature of the substrate and the substrate material had a strong influence on the morphology of the graphitic carbon nanowall structures. With regard to these results, a first growth model for the deposition of CNWs by ICP-PECVD and aluminium acetylacetonate is proposed. This model explains the formation of four different morphologies (nanorods as well as thorny, straight and curled CNWs) by taking the surface diffusion into account. The surface diffusion depends on the particle energies and the substrate material and thus explains the influence of these parameters.

## Introduction

The first report of the synthesis of carbon nanowalls (CNWs), i.e., wall-like carbon nanosheets aligned perpendicular to the substrate, was given by Wu and co-workers [1]. The average thickness of these sheets was measured by using transmission electron microscopy to be of several nanometers [2–7], similar to that of vertically aligned carbon nanotube arrays [8]. In CNWs, few graphene layers stick together like thin graphite flakes. The height of the CNWs can be of several micrometers, contributing to the large surface area of the material. The high aspect ratio together with chemical stability, mechanical strength and electrical conductivity make CNWs an interesting matrix material for catalytic applications. Together with metallic nanoparticles, such as platinum, CNWs could be used in lithium-ion batteries, electrochemical sensors or fuel cells [3,9–15]. Due to the high aspect ratio and the sharp top edges of the CNWs, a possible application could also be seen as electron field emitters [16]. Depending on the chosen deposition parameters, CNWs can have superhydrophobic or superhydrophilic properties, which has a significant effect on cell growth, making CNWs an interesting material for biotechnology (bio-sensors) and medical technology (implants, diagnostics) [13,17].

**Figure 1:**
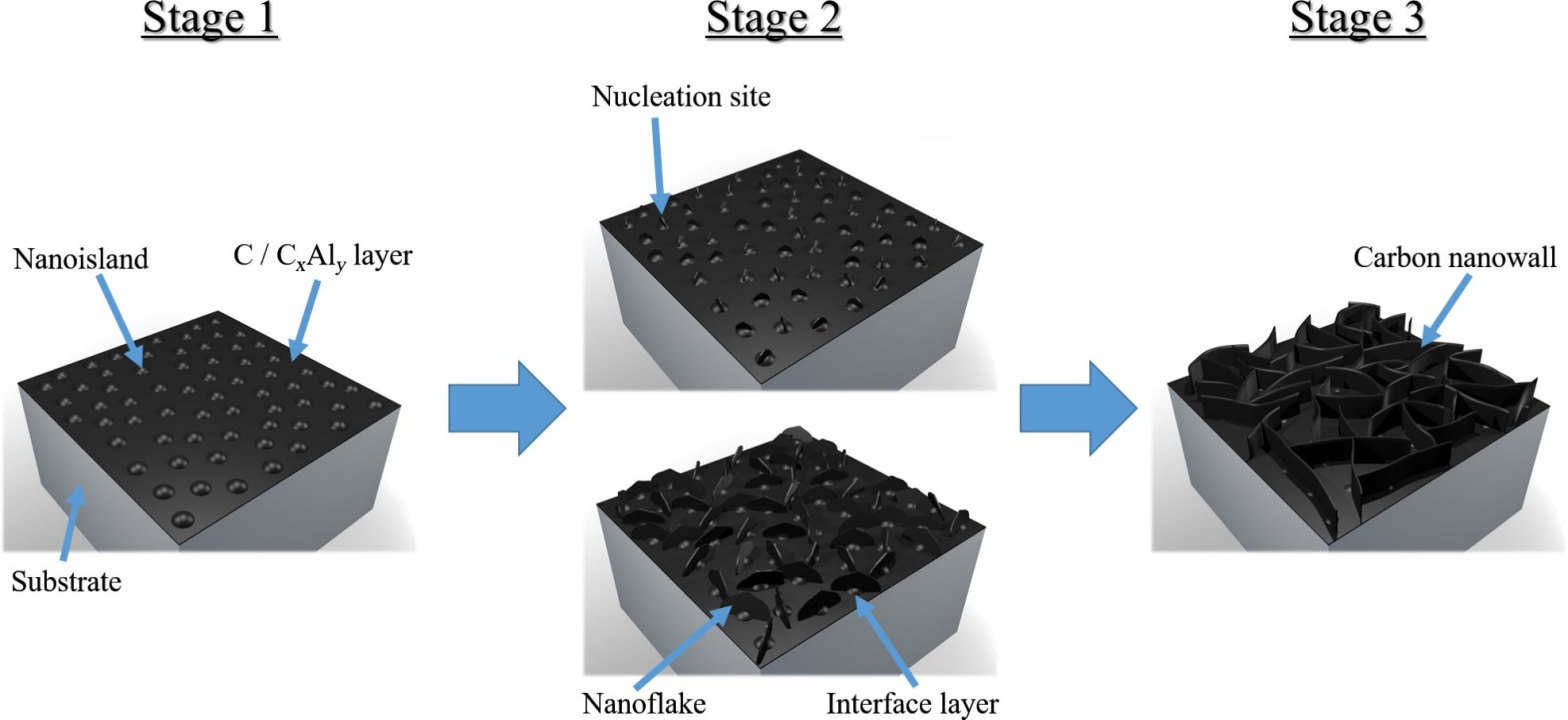
Three-stage model of CNW growth (adapted from Kondo and co-workers) [18].

The growth of CNWs is often referred to as a three-stage process that was originally proposed by Kondo and co-workers [18]. This growth model can be adapted to the synthesis from the metal-organic precursor (aluminium acetylacetonate, C_15_H_21_AlO_6_) used in this work (Figure 1).

At the beginning of the deposition process, a very thin carbon or C*_x_*Al*_y_* layer is deposited onto the substrate. Carbon species are further generated from the gaseous radicals in the plasma and condensate on the surface to form nanoislands with dangling bonds as described in the Stranski–Krastanov growth model (stage 1). In stage 2, a high density of carbon nanoislands is reached on the substrate, offering a rough surface with a considerable number of dangling bonds that act as nucleation sites for randomly orientated carbon nanoflakes. 2D growth and the subsequent formation of (few-layer) graphene sheets follow. The nanoflakes being almost vertically aligned on the substrate grow fastest to finally form vertically standing nanosheets. The reason for this preferred growth is the higher field strength in the plasma at the exposed edges of these CNWs. Thus, the smaller inclined nanoflakes are overshadowed by the faster growing nanowalls, ultimately terminating the growth of the smaller ones resulting in a film with perfectly vertically aligned nanowalls. At this point, the nanoflakes grow in height and length and build the typical films consisting of a network of carbon walls [18]. In this later growth stage the influence of the substrate material on the growth is neglectable [19].

An alternative growth model suggested by Cheng and Teii is based on so-called crowding effects [20]. They suggest that horizontally aligned CNWs first grow until they touch each other and, due to the lack of space, later raise and grow vertically. Zhu et al. used polycrystalline silicon substrates and proposed that after synthesizing an initial graphene layer on the substrate, the vertical growth originates at cracks that occur at grain boundaries of the graphene that are rolling up [21]. All these works show that the substrate material can have a significant influence on the formation of the CNWs. Malesevic et al. [22] took a similar approach also proposing that the initial graphene rolls up at defects. They suggested that these defects are due to ion bombardment in the PECVD process as well as due to temperature gradients on the substrate. This is a first indication for how the particle energy of the plasma species could influence the resulting CNW structure. Often, the CNW growth is explained simply by the Vollmer–Weber growth model, with the growth direction changing from horizontal to vertical as soon as the substrate is fully covered [9].

With a few exceptions of using metal-organic precursors [20] or graphite, in the majority of experiments for CNW deposition in literature gaseous precursors have been used as carbon source. Gases used are typically methane (CH_4_), acetylene (C_2_H_2_) and hexafluoroethane (C_2_F_6_) mixed with argon or hydrogen as carrier gas.

The synthesis from the solid metal-organic precursor Al(acac)_3_ was up to now only reported by our group [23–24]. The advantage of this solid precursor is that the CNW are deposited from a single-source metal-organic precursor providing the right composition of the process gas as well as offering potential metallic components that can be built into the carbon matrix as functional material as shown with aluminium [20]. The ICP-PECVD source features a special antenna design, which provides the possibility to vary particle flow and particle energy independent of each other. Furthermore, energies can be set to very low levels (down to plasma energy) [23].

## Experimental

### PE-CVD of CNWs

**Figure 2:**
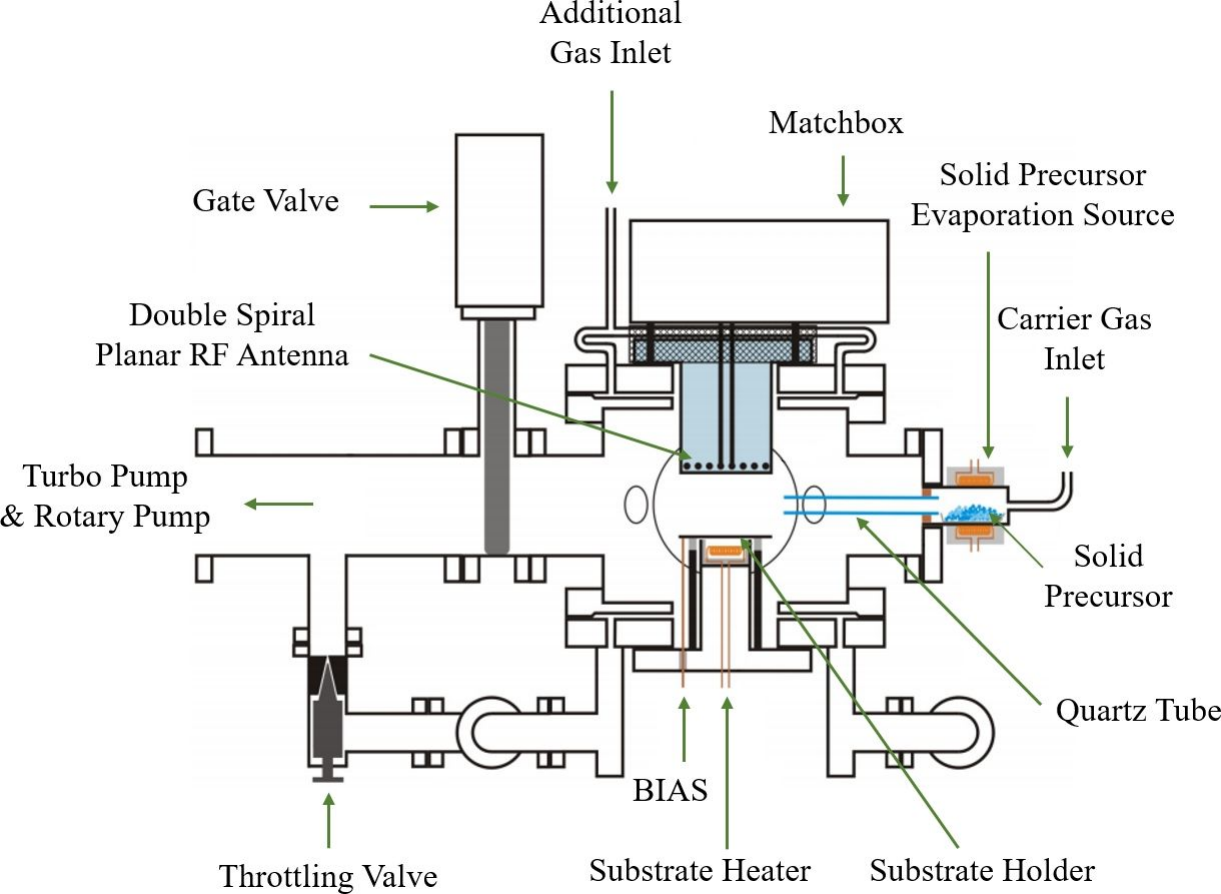
Schematic diagram of ICP-PECVD plasma source.

CNWs were deposited from aluminium acetylacetonate (Al(acac)_3_) as precursor in an ICP-PECVD reactor. A schematic diagram of the inductively coupled plasma system is shown in Figure 2. The plasma is generated in a gaseous electronics conference (GEC) reference cell reactor [25] with a special modified inductively coupled plasma antenna allowing for high plasma densities even at low particle energies [26].

The Al(acac)_3_ precursor in powder form was sublimated in a fluidized bed evaporator at a constant temperature of 127 °C and then transported into the reaction chamber by using argon as carrier gas. According to the vapor-pressure curve taken from Siddiqi et al. and a publication from Nielsen et al. [27–28] the precursor flow can be estimated to be about 1.66 sccm. The precursor flow and the flow of the Argon carrier gas (40 sccm) were kept constant for all experiments. Depositions were performed at a pressure of 8 Pa and the deposition time was 50 min for all films shown here. The plasma power was kept constant at 500 W. The distance between the RF antenna and the substrate is 100 mm. Hence, the substrate is directly exposed to the plasma.

We investigated the influence of the substrate material, the substrate bias and the substrate temperature on the deposition process and on the structure of the synthesized CNWs. Four different kinds of substrates were chosen: stainless steel, aluminium, nickel and silicon. These materials were chosen because they differ significantly in essential properties such as carbon solubility, and it was expected, that this should have a strong influence on the nucleation and growth process of the CNWs.

Three different substrate temperatures were used to investigate the influence on the growth process (350, 425 and 500 °C). Furthermore, the bias voltage was set to values of 0 V (GND), −10 V, −30 V and −100 V (bipolar pulse: 5 ms negative; 0.5 ms positive).

### Characterization of nanowalls

The morphology and the chemical composition of the films were measured by scanning electron microscopy and energy-dispersive X-ray spectroscopy (Quanta 400 FEG). For the evaluation of the wall length and height from the SEM images an open-source software (ImageJ, National Institute of Health) was used. A scanning Auger Nanoprobe (Ulvac-Phi 710) was used to perform chemical mappings of the cross sections of the films. Raman spectroscopy is used as a non-destructive analytical tool to measure the structure of the carbon bonds in the materials. In this work the method is used to determine the sp^2^/sp^3^ ratio, the disorder of the carbon structures [29–30] and to get spectroscopic fingerprints for the different structures described before. The Raman spectra in this paper are obtained by a Renishaw inVia REFLEX Raman spectrometer with a 633 nm (1.96 eV) laser. The laser power was set to 1% and the exposure time was 10 s. Raman spectra were taken at three different positions in a range from 900 to 3200 cm^−1^ for each sample and the individual measurements were averaged and smoothed for the evaluation.

## Results and Discussion

### Morphology

**Figure 3:**
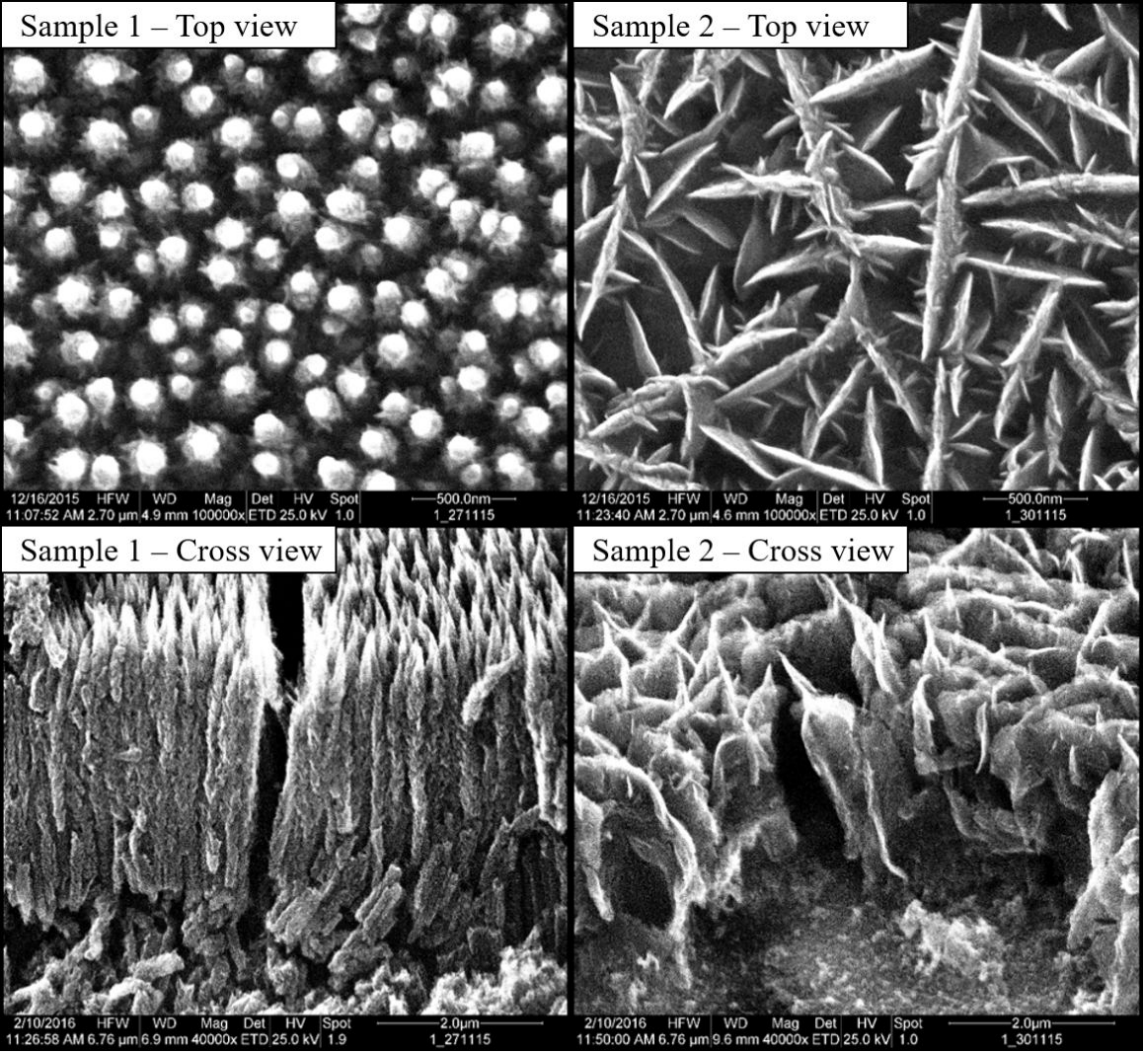
SEM images. Sampe 1 deposited at 350 °C and 0 V bias voltage: nanorods; sample 2 deposited at 350 °C and −10 V bias voltage: thorny CNWs. All grown on stainless steel substrates. Cross-section images taken under from an angle of 45°.

**Figure 4:**
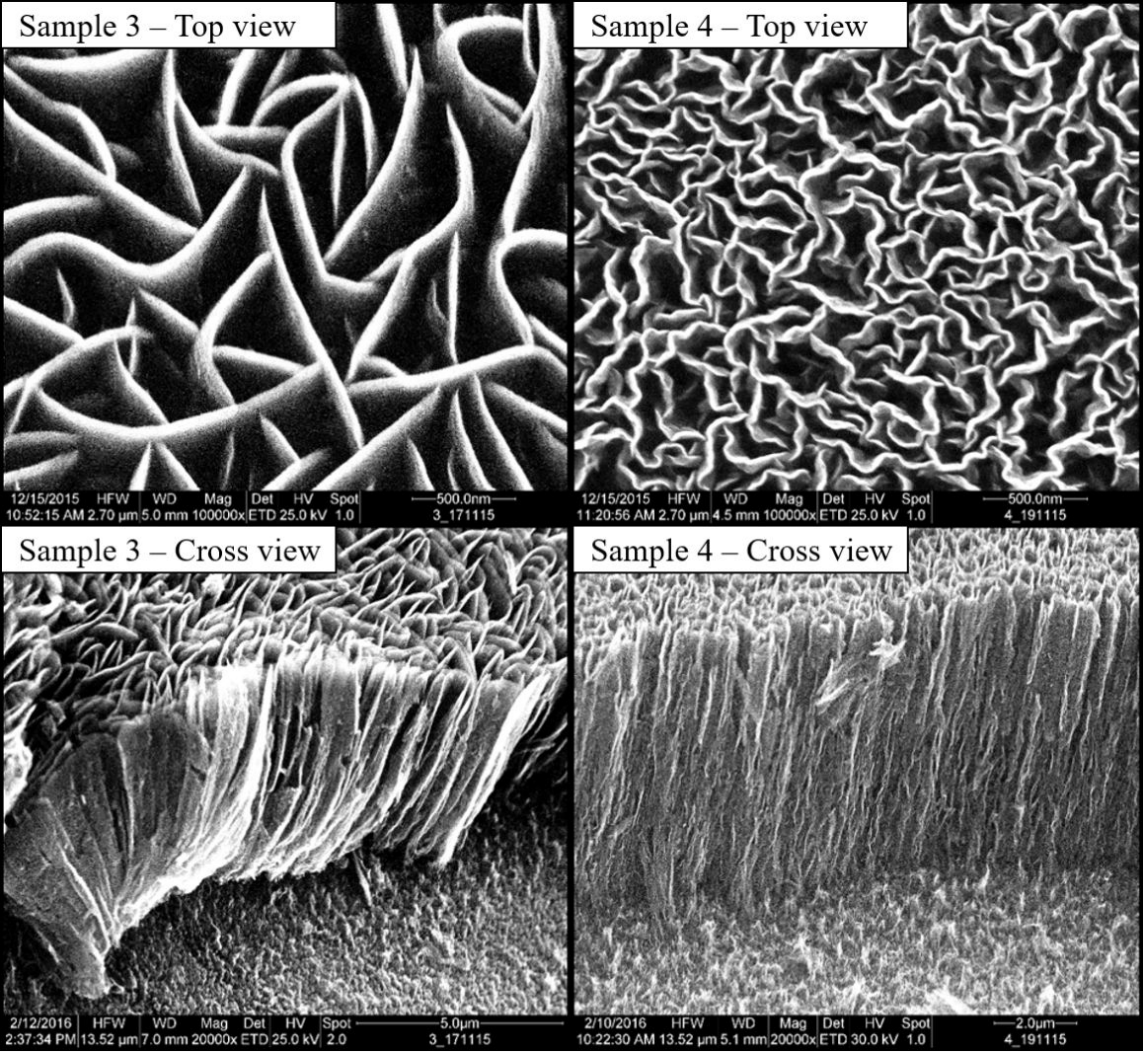
SEM images. Sample 3 deposited at 425 °C and −30 V bias voltage: straight CNWs; sample 2 deposited at 500 °C and 0 V bias voltage: curled CNWs. All grown on silicon substrates. Cross-section images taken under from an angle of 45°.

Figure 3 and Figure 4 show the morphology of the films obtained in the given parameter range. Both pictures show the top-view of the samples in the upper row and the corresponding cross sections of the films at the bottom row. The morphologies can be classified into four major classes: nanorods (Figure 3, sample 1), thorny structures (Figure 3, sample 2), straight CNWs (Figure 4, sample 3) and curled CNWs (Figure 4, sample 4).

The mean wall length was measured to be between 127 and 391 nm for the curled CNWs and between 507 and 1152 nm for the straight CNWs. Beside the carbon wall length, the density of CNWs per area was obtained from the SEM measurements of the samples. The density was calculated to be between 13 and 130 µm^−2^ for the curled CNWs and between 1.3 and 7.7 µm^−2^ for the straight CNWs adding to the large surface area of the material.

Although the thickness of the CNWs was not systematically measured in this work, it is clearly visible from the SEM images that the thickness is also varying with the structure. Nanorods and thorny CNWs show a higher thickness compared to the straight and the curled CNWs. The latter feature a thickness down to a few nanometers. The top-view SEM images overestimate the thickness due to the slightly curled tips of the CNWs that show a bright contrast in the image. However, the cross sections show that in fact the thickness is comparable to the CNWs described in the literature.

**Figure 5:**
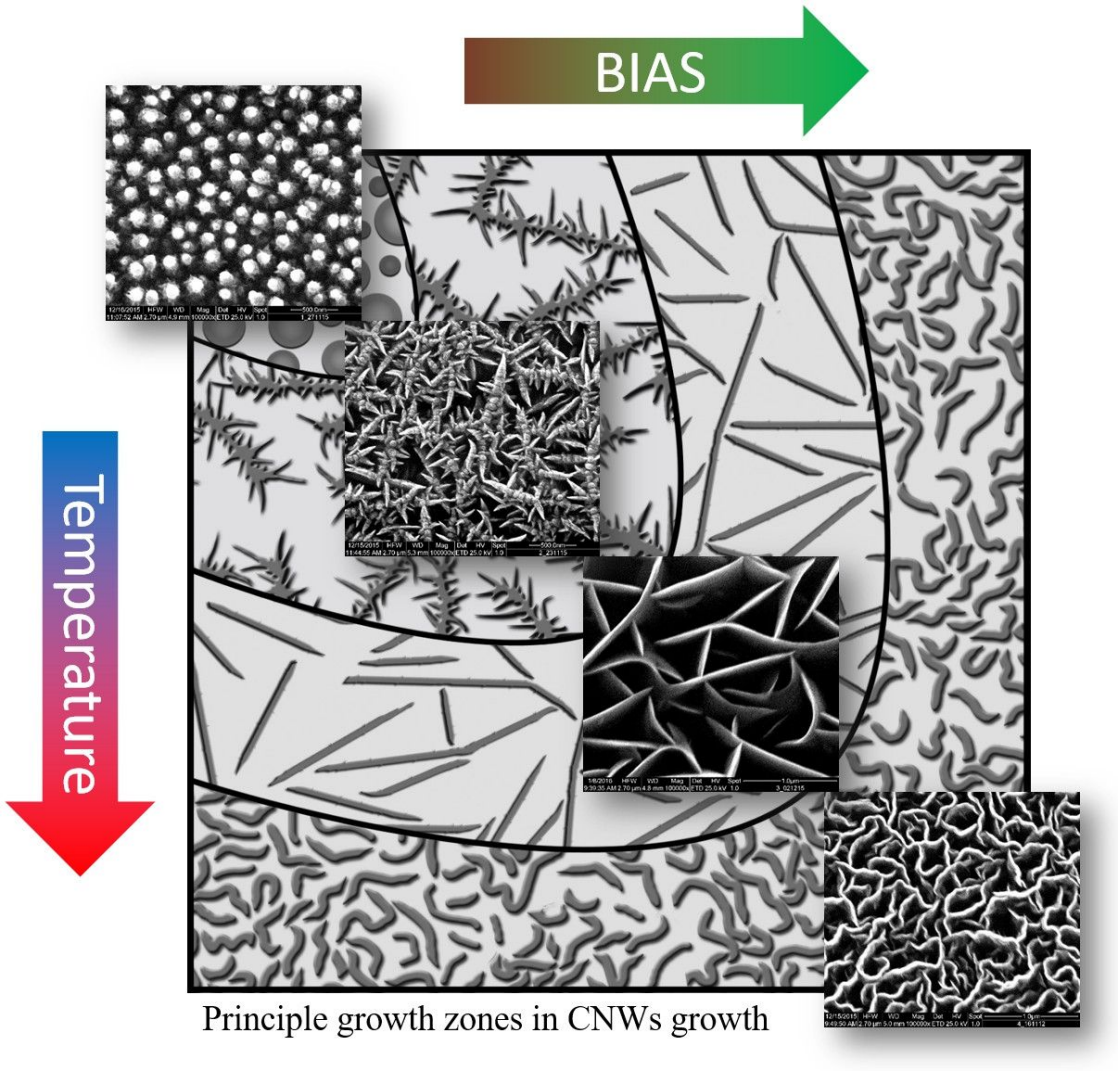
Growth zones of CNW growth: nanorods (low energies), thorny structures and straight CNWs (medium energies) and curled CNWs (high energies).

The growth of the specific structures can be controlled through the bias voltage and substrate temperature, both parameters that influence the particle energy of the growth precursors. The dependence is indicated schematically in Figure 5. By increasing bias voltage and substrate temperature the growth zones are changed from nanorods at low energies, to thorny structures and straight CNWs at medium energies and curled CNWs at high energies.

**Figure 6:**
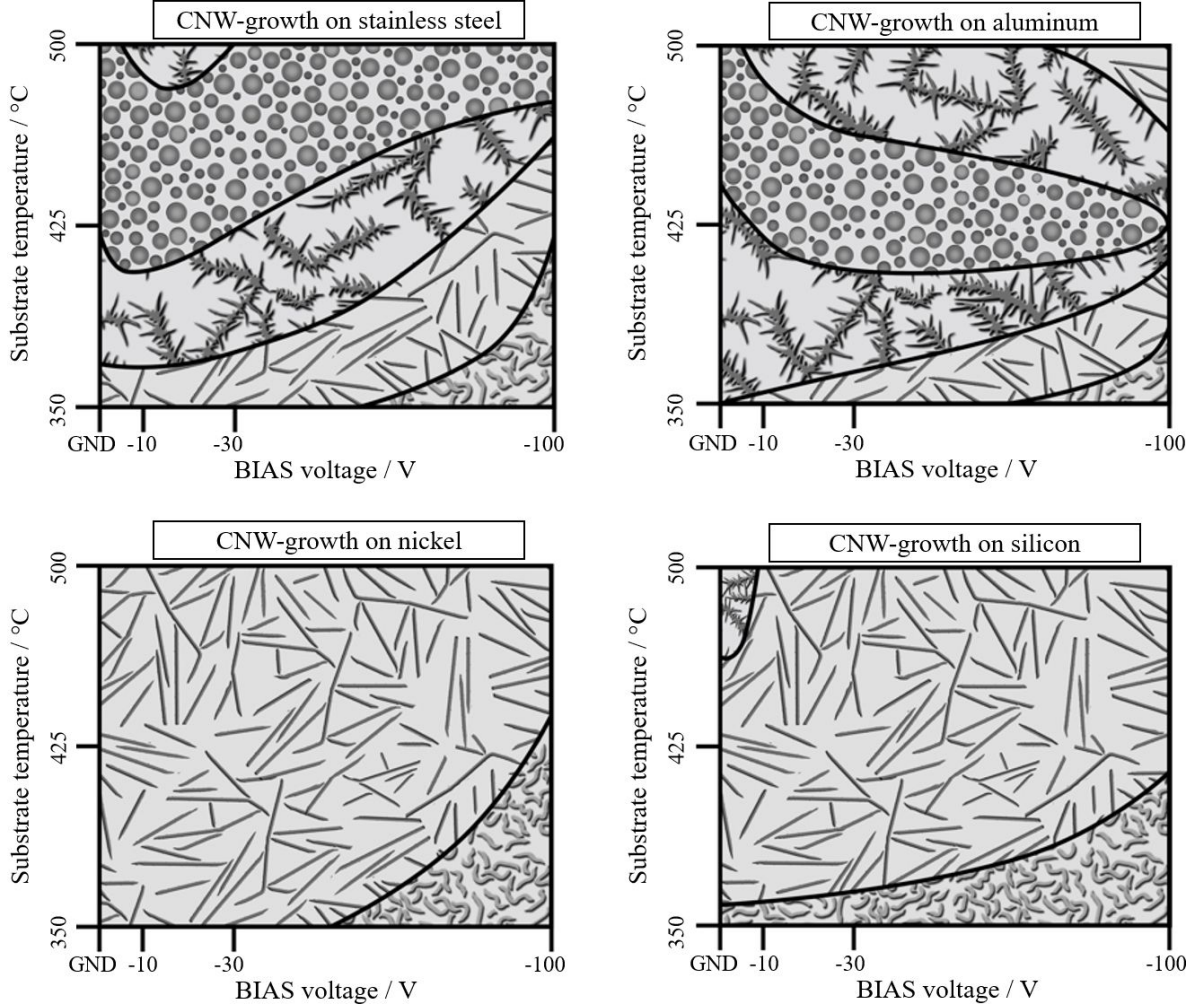
Growth zones on different substrate materials as a function of substrate temperature and bias voltage.

The influence of four different substrate materials on the CNW structure was also investigated. The growth zones for these substrates are shown schematically in Figure 6. It was found that the described growth model is also valid for all of the chosen materials. However, the growth regimes are shifted for the different materials. While all of the above mentioned structures are found on stainless steel and aluminium (although shifted to different bias voltage and temperature values), on nickel and silicon no nanorods growth zones could be identified and only straight and curled CNWs were found. The explanation for this observation is possibly the difference in carbon bulk and surface diffusion for the given materials and the different affinities to form carbide at the surface.

Surface diffusion can generally be considered as particles moving between adjacent adsorption sites on a surface. This motion strongly depends on the energy of the particle at the surface, typically increasing with increasing temperature, as well as on the various surface properties influencing the interplay of the adsorbed particles and the surface [18–19,31]. At relatively low energies (low bias voltage and low substrate temperatures), we observe the formation of nanorods. From SEM images (Figure 3) one can see that the diameter of these rods is rather large (about 100 nm), that they are growing tightly next to each other on the surface and that they are of dense structure. Due to their low energy, the growth species cannot migrate far on the substrate surface and are therefore incorporated into the rather dense graphitic structure of the carbon nanorods. At higher energies, the diffusion length for the growth species at the surface is larger and nucleation occurs more separated in space (similar to the Volmer–Weber growth). Nanoflakes can grow subsequently from these nucleation sites leading to the formation of straight CNWs. This process is similar to the model suggested by Kondo and co-workers [18]. A further increase of the particle energy leads to the synthesis of curled CNWs due to a higher nucleation density at the surface. The higher nucleation density results in a higher CNW density per area and thinner walls compared to the straight CNWs.

Apparently, there are two effects at play. First, a higher particle energy leads to a higher surface diffusion and thus less nucleation sites for the CNWs. However, if we increase the energy of the growth species even higher by increasing substrate temperature and the bias more defects are induced in the initial carbon growth layer. It was described before that defects in initial graphene and graphite layers can lead to grain boundaries and defects that fold up and can be seen as additional nucleation sites for the growth of CNWs [21]. This model of increasing defects in the CNWs is also backed by Raman spectroscopy measurements as shown below. Thus, the nucleation and growth process is influenced by these two effects, which are competing against each other, resulting in the different structures depending on the energy of the growth species. The thorny CNWs that can be found at rather low energies can be understood as transitional form between the nanorods and the straight CNWs.

### Chemical characterization

**Figure 7:**
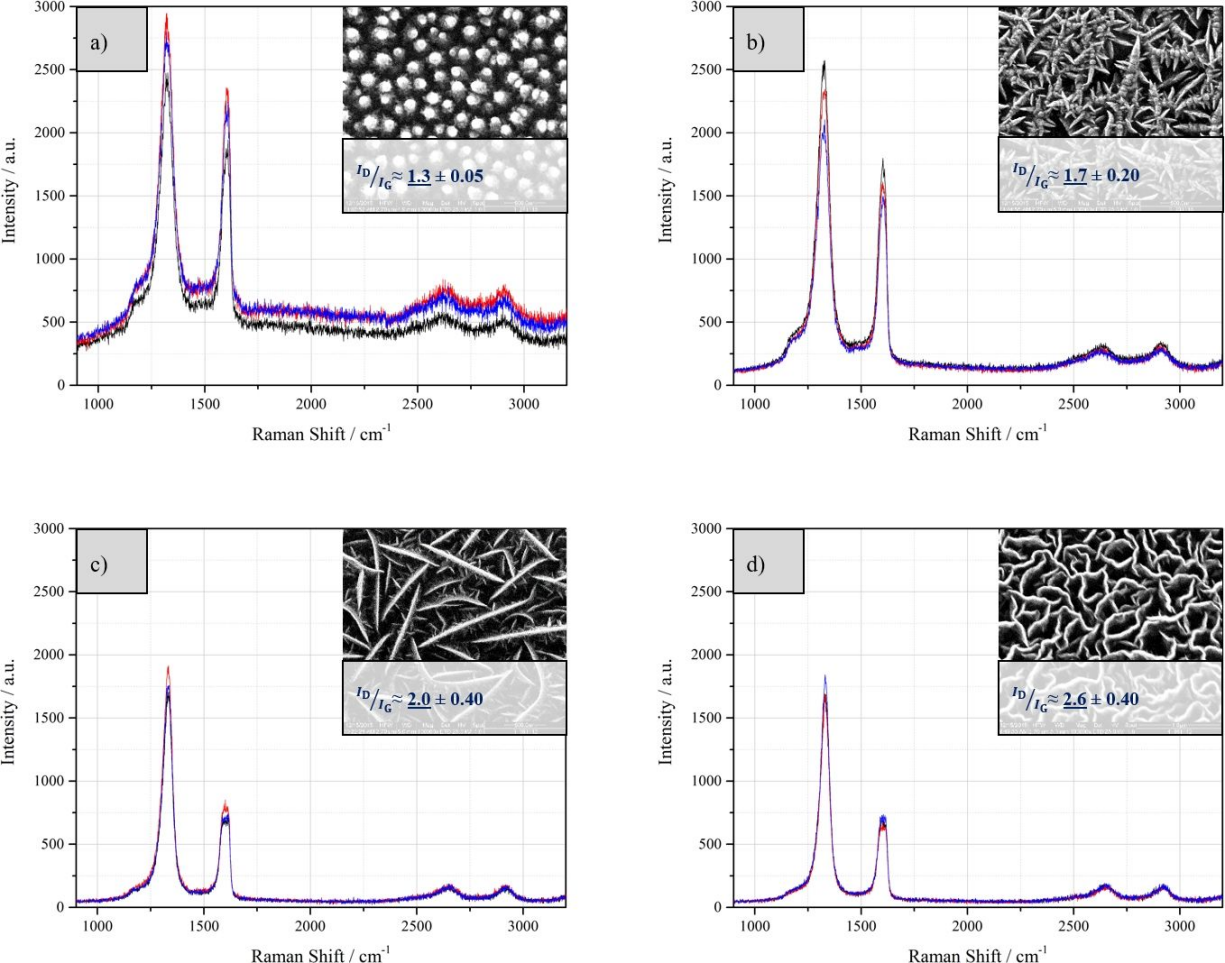
Typical Raman spectra and the *I*_D_/*I*_G_ ratios for a) nanorods, b) thorny CNWs, c) straight CNWs and d) curled CNWs. All structures were synthesized on stainless steel.

Raman spectroscopy can give information about the hybridization and the defect density of the carbon material. All samples were measured at three different positions and the measured values were averaged for the interpretation. In Figure 7, typical Raman spectra are shown for the respective structures. Although the intensities of the Raman peaks differ, the typical carbon peaks were measured in all of the samples. A strong G-peak is found with the position slightly shifting from 1582.87 to 1616.19 cm^−1^ for the samples shown in this work. Additionally, a D-peak is found that is typical for CNWs with positions measured between 1319.66 and 1333.89 cm^−1^ in our samples.

In comparison to earlier publications describing CNWs from the plasma process, the Raman spectra shown here have a significant lower D′-band intensity at 1615 cm^−1^ which is associated with lattice defects of different nature [32–33].

**Figure 8:**
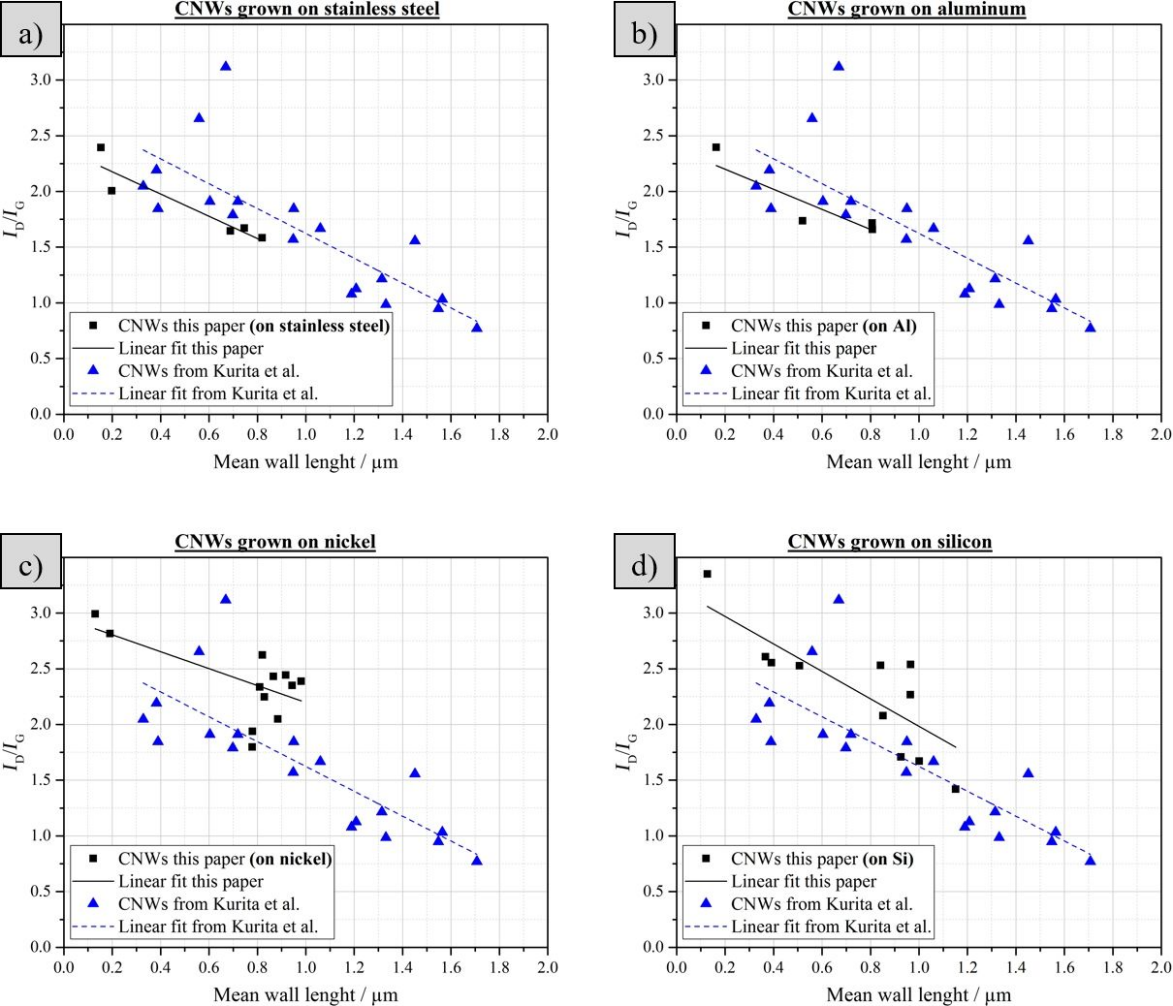
*I*_D_/*I*_G_ ratio as a function of the mean wall length of the straight and the curled CNWs on a) stainless steel, b) aluminium, c) nickel and d) silicon; blue triangles: data points taken from [34].

A significant difference was found in the samples with respect to the intensities of the measured D- and G-peaks. Raman spectroscopy of the four different morphologies shows that a specific *I*_D_/*I*_G_ ratio can be assigned to each structure. The value increases from 1.3 ± 0.05 for the carbon nanorods, over 1.7 ± 0.2 for the thorny structures and 2.0 ± 0.4 for the straight CNWs to 2.6 ± 0.4 for the curled CNWs. It is thus shown that the particle energy also has influence on the structural composition of the nanowalls as measured with the Raman spectrometer. The higher the particle energy the higher the *I*_D_/*I*_G_ ratio measured for the resulting CNWs. The difference in the *I*_D_/*I*_G_ ratio between the straight and the curled CNWs can be attributed to the different mean wall lengths. Kurita et al. showed [34] that there is a correlation between the *I*_D_/*I*_G_ ratio and the wall length and we compared our measurements with their results. The black squares in Figure 8 represent the *I*_D_/*I*_G_ ratios and the mean wall lengths measured in this work while the blue triangles are the data found by Kurita and co-workers.

The linear fits for the data points of the CNWs synthesized on stainless steel (Figure 8a), aluminium (Figure 8b) and silicon (Figure 8d) show a similar slope compared to the data from Kurita et al. (quartz substrates with different catalytic layers, 532 nm, 0.5–3.0 µm), while the data points for the CNWs on nickel as substrate show a slightly smaller slope. The *I*_D_/*I*_G_ ratios of the different structures synthesized in this paper are in good agreement with the results from previous publications and shows that the material is in fact comparable with the other works on CNWs.

The intensity of the disordered peak (and thus the *I*_D_/*I*_G_ ratio) is higher for shorter mean wall lengths. A high intensity of the D-peak can be interpreted as high defect density in the carbon material. Since shorter walls mean more edges and smaller graphitic crystals, the defect density is higher with more carbon atoms at grain boundaries. Furthermore, the specific surface of the material is higher leading to more dangling bonds at the surface of the CNWs.

By taking the values measured by Raman spectroscopy, the inner structures of the CNWs can further be classified with respect to the three-stage model of Robertson and co-workers [35]. By applying the minimal and the maximal value for the G-peak position measured by Raman spectroscopy to this model the synthesized structures can be characterized as graphitic or nanographitic material. The minimal and maximal *I*_D_/*I*_G_ ratios measured in the samples suggest that the material is nanographitic to slightly amorphous carbon. Combining the two values one can deduce that the synthesized material is almost entirely sp^2^-bonded nanocrystalline graphite with almost no carbon being sp^3^-bonded.

**Figure 9:**
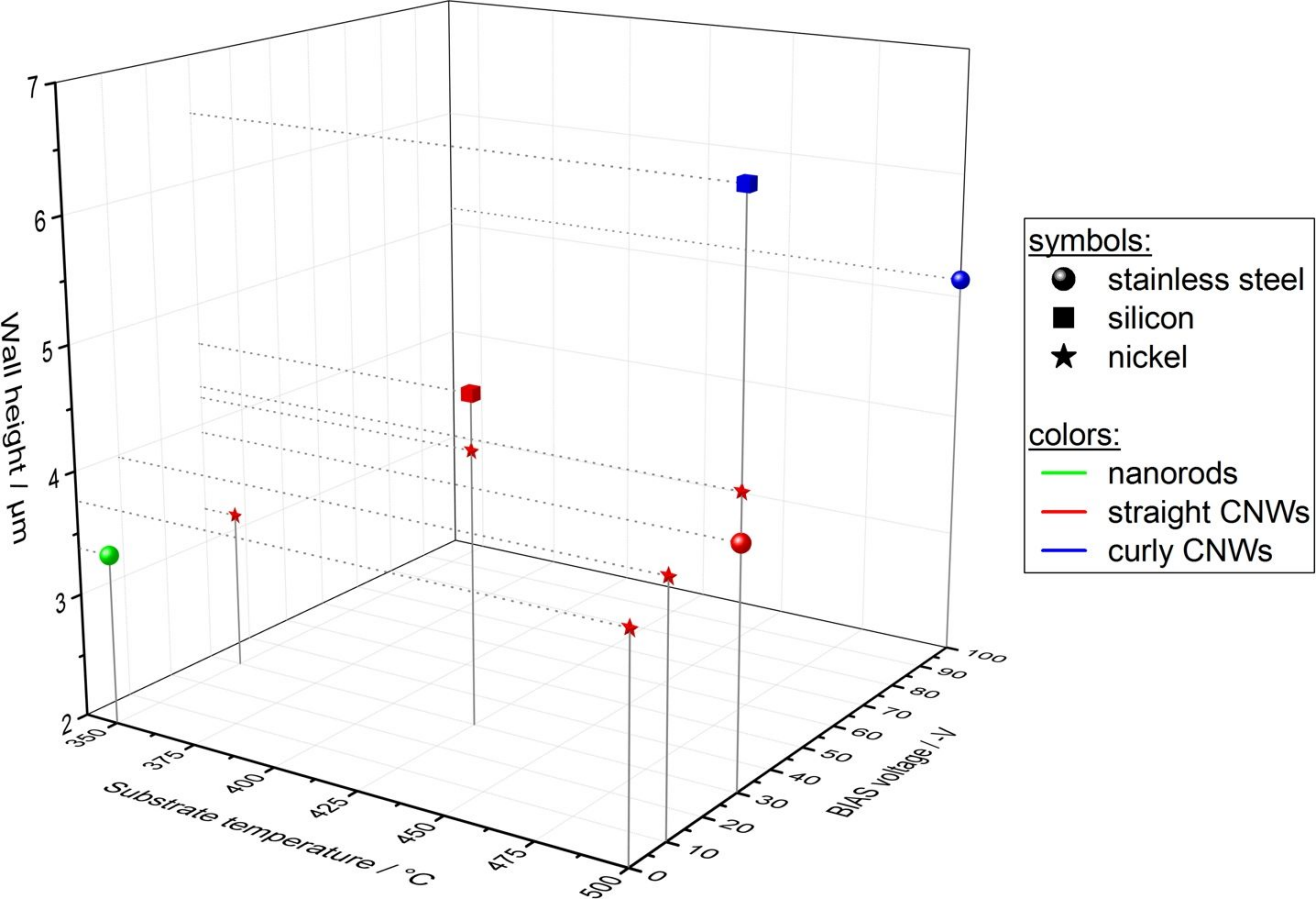
Heights of CNWs as a function of process parameters and substrate material.

The heights of the CNWs were also obtained from the cross-sectional SEM pictures in Figure 3 and Figure 4 and plotted in a diagram shown in Figure 9. Error bars were omitted in this diagram since the measurement errors of temperature, bias and wall height are negligible at this scale.

The shape of the data points in Figure 9 indicate the substrate material (spheres are on stainless steel, cubes are on silicon, stars are on nickel) while the color of the data points represents the structure of the synthesized CNWs (nanorods are green, straight CNWs are red, curled CNWs are blue). On stainless steel, the nanorods (green sphere) are the shortest structures. Since the SEM pictures already showed that the nanorods are the densest of the structures observed here, it can be expected that for the less dense structures (straight and curled CNWs) the walls grow higher. This is in line with the observations with the curled CNWs being the thinnest and thus highest structures observed. The same observation can be made on silicon with the curled CNWs (blue cube) being higher than the straight CNWs (red cube). Looking at the data points one can see that for any of the used substrate materials, a higher bias voltage and a higher substrate temperature always lead to higher CNWs. Just as the particle energy defines the structure of the synthesized CNWs it also influences the heights of the structures with increasing height from the nanorods (smallest) to the thorny structures, to the straight CNWs, to the curled CNWs (highest). The nanorods are the shortest structures since they are rather thick and grow very dense on the substrates. The growth species have low particle energy and the resulting structures are rather compact with relatively low surface area. In contrast to this, the thorny structures already show spaces between the individual walls. The higher particle energy leads to a growth mode where the carbon structures can grow more in height and the surface show less coverage. This trend continues for the straight CNWs and, thus, these walls are even higher. The curled CNWs grow highest, although the surface coverage increases again (76% compared to 60%) for the straight CNWs). This can be explained by the higher nucleation density and the fact that the curled CNWs are much thinner, which, again, leads to higher structures.

**Figure 10:**
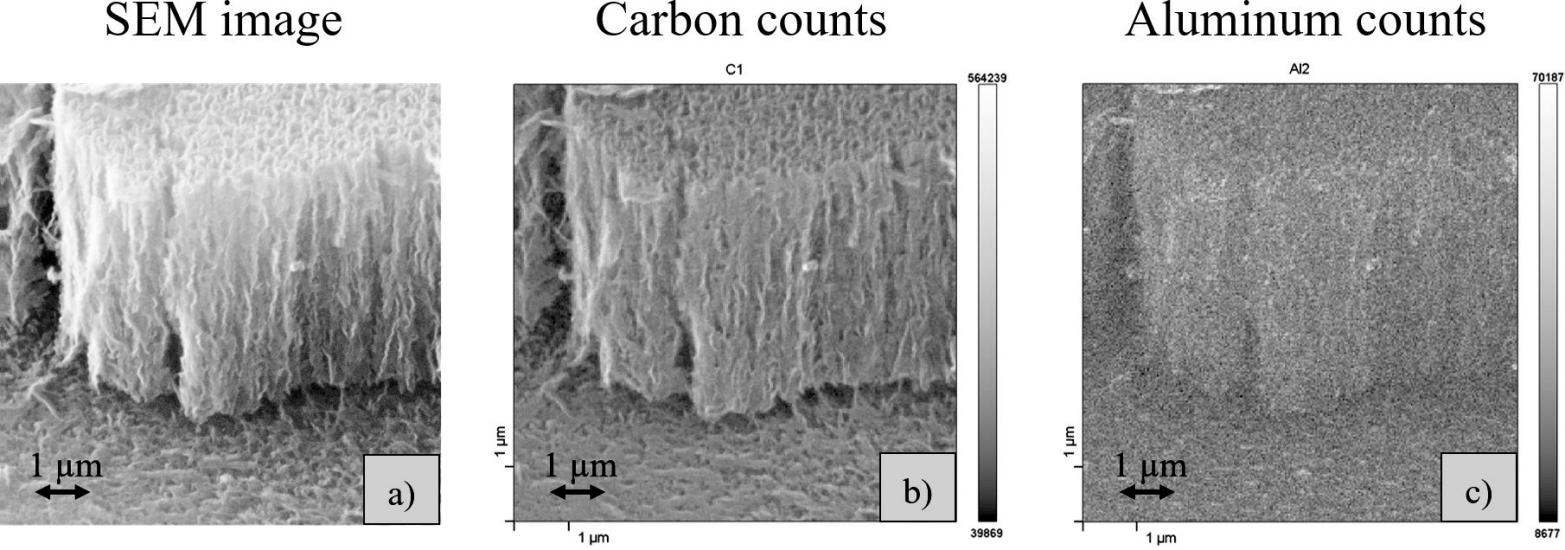
a) SEM image of the curled CNWs and corresponding b) carbon and c) aluminium mappings, as measured by Auger electron spectroscopy.

Scanning Auger electron spectroscopy (AES) was used to obtain the chemical mappings of the cross sections of the films. Since the CNWs are synthesized from an aluminium-containing precursor, the question was whether aluminium can also be found in the CNWs and whether aluminium is accumulated for example at the interface between substrate and CNWs, in the CNWs or at the top of the CNWs. This could provide information about how the aluminium is participating in the growth and/or the nucleation of the CNWs. Figure 10 shows a SEM cross section of a sample with curled CNWs with the corresponding mappings for the distribution of carbon (Figure 10b) and aluminium (Figure 10c) in the film. At every pixel (corresponding to a spot size of 30 nm) an AES spectrum was taken. The mappings show the intensity of the carbon and the aluminium signals as a function of the position. A concentration can be calculated from these intensities. The CNWs mainly consists of carbon and the concentration is calculated to be above 90 atom %. The concentration of aluminium was calculated to be around 6 atom % with an estimated error of ±3%. Since the intensity of the aluminium is much weaker, the morphology is not so clearly represented in the picture as in the carbon measurement where the concentration (and thus the signal) is much higher. No visible accumulation of aluminium can be found in the film or close to the substrate surface. However, the resolution limit of the AES does not give detailed information about how the aluminium is distributed in the carbon matrix. If agglomerations are present in the film, their size must be about the same size as the AES spot of 30 nm or even below. In previous works we already found nanocrystalline Al_3_C_4_ clusters by TEM measurements in our CNWs that were measured to be of 30 nm in size [23].

**Figure 11:**
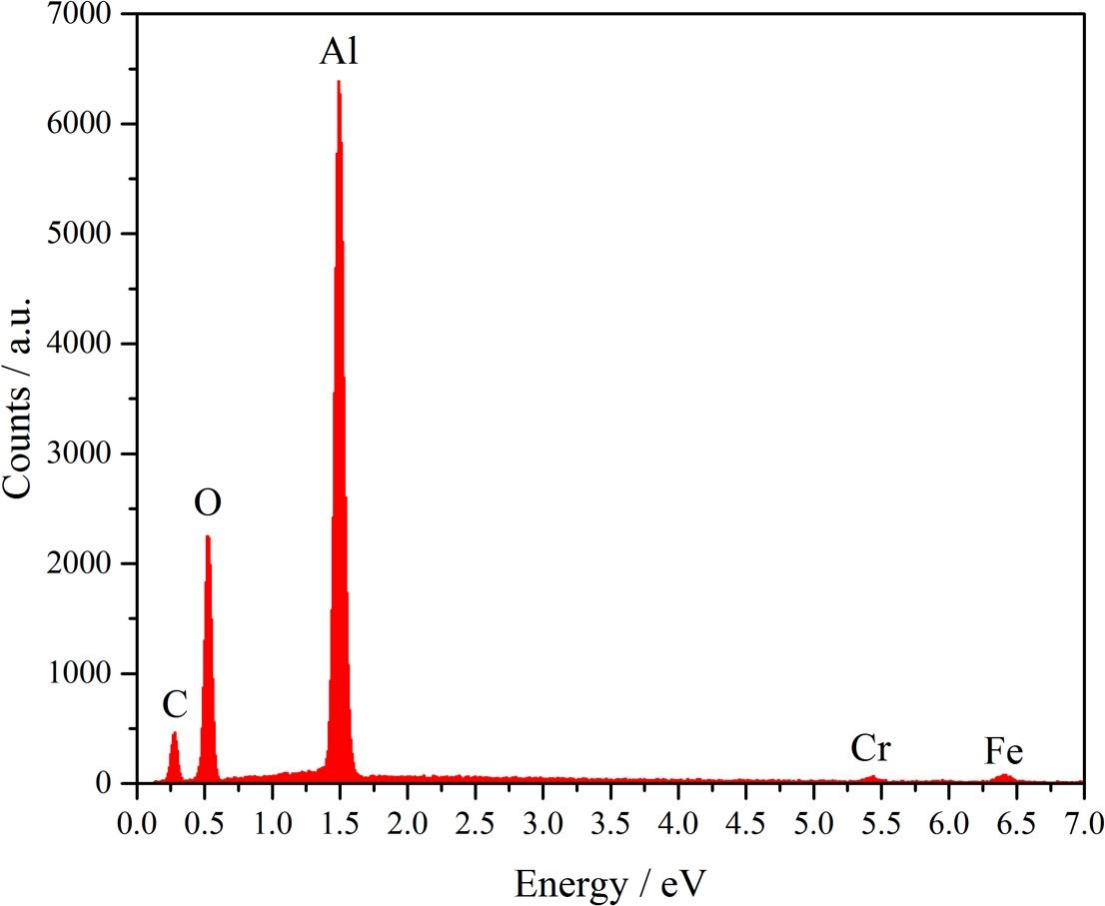
EDX spectrum of CNWs on stainless steel (Fe, Cr). Besides carbon and aluminium, oxygen can be found in the film.

In addition to the AES measurements, EDX measurements were carried out on the CNWs to identify additional trace elements in the carbon structures (Figure 11). Besides the already measured carbon, aluminium, chromium and iron from the stainless steel substrate, and oxygen can be identified. The oxygen is likely from the metal-organic precursor, which also contains oxygen. Thus, in addition to the Al_3_C_4_ clusters, also Al_2_O_3_ compounds in the films are possible, also H_2_O adsorption cannot be excluded.

## Conclusion

Using argon as carrier gas, CNWs were synthesized on aluminium, stainless steel, nickel and silicon substrates from an Al(acac)_3_ precursor. By systematic variation of the substrate temperature (350, 425 and 500 °C) and the bias voltage (0 V (GND), −10 V, −30 V, −100 V), CNWs of very different morphologies were deposited. Four main morphological types were identified by SEM analysis and Raman spectroscopy. It was shown how the combination of substrate temperature and bias voltage determines the resulting morphology. Low temperature and low bias voltage lead to carbon rods. Successively, increasing these values changes the morphology first to a thorny structure and then to straight CNWs. At the highest values of substrate temperature and bias voltage curled CNWs are deposited. The substrate material also has a strong influence on the morphology types that were synthesized. SEM measurements were used to measure the heights and length of the CNWs and these measurements were supported by Raman measurements. The intensity ratio of the D-peak and the G-peak (*I*_D_/*I*_G_) shows a linear relation to the length of the CNWs. It was shown, that higher substrate temperatures and bias voltages lead to structures with higher surface area (thinner walls, higher walls, higher surface densities). Moreover, specific *I*_D_/*I*_G_ ratios could be assigned to the four different morphologies on our samples, giving a fast method to identify and characterize the structures on the samples without the need of more elaborated (and time-consuming) methods such as SEM measurements.

On basis of the experimental results found here, a possible growth mechanism was discussed. This model is based on the model from Kondo et al. and it explains the formation of the four different morphologies by taking the different surface diffusion into account [18]. The surface diffusion depends on the particle energies and the substrate material. In addition, defects in the grown structures are discussed as additional nucleation sites at higher particle energies resulting in the highest density of CNWs on our substrates.

AES and EDX measurements showed an aluminium concentration of around 6 atom % homogenously distributed in the CNW films.

Due to their high surface area the deposited CNWs seem to be an ideal matrix material for catalytic applications. In future works, metallic particles will be deposited on the walls to make them catalytically active and characterize their properties for this application.

Although the nanorods have the smallest surface area of the synthesized morphologies in this work, they might be promising for electron field-emitter applications since they feature nanosized tips. The field-emission properties of these nanorods are also characterized in a future publication.
